# Transitioning from having no metabolic abnormality nor obesity to metabolic impairment in a cohort of apparently healthy adults

**DOI:** 10.1186/s12933-023-01954-w

**Published:** 2023-08-26

**Authors:** Hadas Ben-Assayag, Rafael Y. Brzezinski, Shlomo Berliner, David Zeltser, Itzhak Shapira, Ori Rogowski, Sharon Toker, Roy Eldor, Shani Shenhar-Tsarfaty

**Affiliations:** 1 Department of Internal Medicine “C”, “D” & “E”, Tel Aviv Sourasky Medical Center, 6 Weizmann Street, 64239 Tel Aviv, Israel; 2 Affiliated with Sackler Faculty of Medicine, The Tel Aviv University, Tel Aviv, Israel; 3 Coller School of Management, Tel Aviv University, Tel Aviv, Israel; 4 Diabetes Unit, Institute of Endocrinology, Metabolism and Hypertension, Tel Aviv Sourasky Medical Center, Tel Aviv, Israel; 5 Department of Emergency Medicine, Tel Aviv Sourasky Medical Center, Tel Aviv, Israel

**Keywords:** Metabolic syndrome, Health, Biomarkers, Hypertension, C-reactive protein, Aging

## Abstract

**Introduction:**

The global prevalence of metabolic syndrome and its association with increased morbidity and mortality has been rigorously studied. However, the true prevalence of “metabolic health”, i.e. individuals without any metabolic abnormalities is not clear. Here, we sought to determine the prevalence of “metabolically healthy” individuals and characterize the “transition phase” from metabolic health to development of dysfunction over a follow-up period of 5 years.

**Methods:**

We included 20,507 individuals from the Tel Aviv Sourasky Medical Center Inflammation Survey (TAMCIS) which comprises apparently healthy individuals attending their annual health survey. A second follow-up visit was documented after 4.8 (± 0.6) years. We defined a group of metabolically healthy participants without metabolic abnormalities nor obesity and compared their characteristics and change in biomarkers over time to participants who developed metabolic impairment on their follow-up visit. The intersections of all metabolic syndrome components and elevated high sensitivity C-reactive protein (hs-CRP) were also analyzed.

**Results:**

A quarter of the cohort (5379 individuals, (26.2%) did not fulfill any metabolic syndrome criteria during their baseline visit. A total of 985 individuals (12.7% of returning participants) developed metabolic criteria over time with hypertension being the most prevalent component to develop among these participants. Individuals that became metabolically impaired over time demonstrated increased overlap between metabolic syndrome criteria and elevated hs-CRP levels. The group that became metabolically impaired over time also presented higher delta values of WBC, RBC, liver biomarkers, and uric acid compared with participants who were consistently metabolically impaired. LDL-C (low-density lipoprotein cholesterol) delta levels were similar.

**Conclusions:**

Roughly one-quarter of apparently healthy adults are defined as “metabolically healthy” according to current definitions. The transition from health to metabolic dysfunction is accompanied with active inflammation and several non-metabolic syndrome biomarkers. Aggressive screening for these biomarkers, blood pressure and hs-CRP might help identify apparently healthy individuals at increased risk of developing metabolic syndrome over time.

**Supplementary Information:**

The online version contains supplementary material available at 10.1186/s12933-023-01954-w.

## Introduction

The metabolic syndrome (MetS) is a-well studied condition which is associated with an increased risk for diabetes and cardiovascular disease (CVD) [1]. While having multiple definitions from different societies and in different populations, the syndrome usually comprises a combination of the following metabolic derangements: high fasting glucose levels, hypertension, low HDL-C (high-density lipoprotein cholesterol) levels, high triglycerides levels and an increased waist circumference [2].

Although the prevalence of MetS and its components have been extensively documented in large populations [3, 4], and the changes in MetS status and its subsequent risks were previously described [5, 6], the rates and characteristics of metabolically healthy individuals (i.e. free of any metabolic abnormalities), especially when stable over time, are rarely mentioned. We note that there is an abundance of studies regarding metabolically healthy obesity (MHO), i.e. obesity as a single metabolic abnormality. However, a recent prospective study has stated that MHO individuals were at higher risk of all-cause mortality, diabetes, CVD, heart failure and respiratory disease compared to metabolically healthy people without obesity [7]. Nonetheless, our group has recently shown that overweight and obese individuals in an apparently healthy population are associated with significant blood tests abnormalities [8]. It is also noteworthy to mention that metabolic health was found to be a more important determinant for the development of diabetes than obesity [9].

A recent prospective study by Guembe et al. [10] has found that individuals with MetS or one of its single components present major cardiovascular events, cardiovascular mortality and all-cause mortality earlier than individuals without MetS. Sinning et al. has recently provided evidence that glycohemoglobin (HemA1C) is independently linked to cardiovascular mortality, overall mortality, and CVD in the general European population [11]. Wang et al. referred to the relevance of triglycerides and fasting glucose by stating that elevated cumulative average triglyceride-glucose index independently predicts ischemic stroke in the general population [12]. Moreover, recovery from MetS was previously suggested to be associated with decreased risk for major cardiovascular events [13]. These findings emphasize the importance of early detection and prevention of metabolic morbidity by differing it from metabolic health and allows us to identify the unique biomarkers that predict new onset of metabolic disorders. Not long ago, Kouvari et al. had published that the baseline presence of non-alcoholic fatty liver disease (NAFLD) acted as a predictor for the development of metabolically unhealthy status and an elevated cardiometabolic risk overtime [14]. We thus sought to assess the prevalence of metabolic health (i.e., the absence of any of the defining characteristics of the metabolic syndrome and diagnosed diseases) in a large cohort of adult individuals, describe its natural trajectory (i.e. development over time) and identify parameters (e.g. liver enzymes, blood count, electrolytes and lipids) that may predict development of future metabolic derangements or even identify current hidden ones.

## Methods

### Study population

Our data was collected between November 2001 and March 2022 at the Tel Aviv Sourasky Medical Center Inflammation Survey (TAMCIS), a registered databank of the Israeli ministry of justice, comprised of a large cohort of apparently healthy subjects. Our participants were employed individuals who attended the medical center for an annual routine check-up and gave their written informed consent for participation in the study. The checkups included an interview with a physician, a physical examination, urine and blood tests analysis, an exercise stress test, and a spirometry test. Thirty eight percent of the individuals returned for a follow-up visit within 5 years.

### Study procedures

Participants were recruited by an interviewer upon arrival to the medical center. First, overnight fasting blood samples were collected, followed by a brief medical history and drug therapy reports. Then physical examinations were conducted by physicians and nurses. The blood samples were centrifuged for 10 min at 3000 rpm at 14 ℃ to obtain the serum. Enzymatic methods were used to assess the serum concentration of total cholesterol, HDL-C, LDL-C and triglycerides (Roche, Mannheim, Germany). Hs-CRP concentrations were determined using the Boering BN II Nephelometer (DADE Boering, Marburg, Germany). For details regarding enzymatic methods to measure fasting plasma glucose and hemA1C, methods of measuring blood pressure or methods of measuring body mass index (BMI) see references [15]. Waist circumference (WC) was measured at the midpoint between the last palpable rib and the iliac crest, using an inelastic metric tape.

### Metabolic syndrome criteria

Definitions of elevated glucose levels, high waist circumference, hypertension, elevated triglycerides and low levels of HDL-C were in accordance with the international harmonized criteria definitions [2]. High glucose levels were defined as fasting plasma glucose (FPG)$\ge$ 100 mg/dL or drug treatment for elevated blood glucose. Abdominal obesity was defined as waist circumference (WC) $\ge$ 102 cm (cm) in males and WC $\ge$ 88 cm in females. Hypertension was defined as systolic blood pressure $\ge$ 130 mm of mercury (mmHg), or diastolic blood pressure $\ge$ 85 mmHg, or treatment with antihypertensive medications. High levels of triglycerides were defined as above 150 mg\dL, or treatment with fibrates or niacin. Low levels of HDL-C were defined as under 40 mg/dL for men and under 50 mg/dL for women treatment with cholesterol lowering medication.

### Additional metabolic factors

In addition, we assessed the levels of elevated Hs-CRP, prediabetes and obesity. These factors were individually associated with an abnormal metabolic state. High hs-CRP, an highly associated inflammatory biomarker with metabolic abnormality [16], was considered elevated if above 3.00 mg\L [17]. Prediabetes, dysglycemia as a precedent condition of diabetes, was defined according to the American diabetes association (ADA) definition, 100 ≤ FPG ≤ 125 mg/dL or 5.7 ≤ HemA1C ≤ 6.5 percent [18]. Lastly, high BMI was defined as above 30 kg/${m}^{2}$ according to WHO [19].

### Definition of baseline metabolic health and impairment over time

**Fig. 1:**
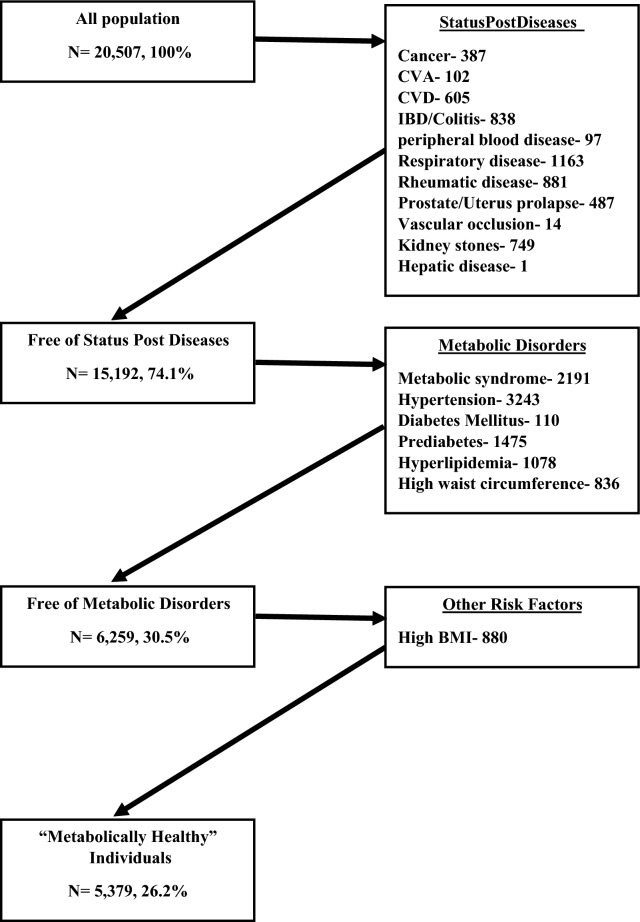
Exclusion flow chart of unhealthy individuals

We defined “metabolically healthy” individuals, as participants without any diagnosed co-morbidities or any metabolic abnormalities, including obesity. Figure 1 shows the exclusion criteria for metabolic health in the entire cohort. A total of 15,128 participants were considered metabolically impaired accordingly. First, we excluded 5315 participants with the concomitant diagnoses: cancer, Cerebro-vascular accident (CVA), CVD, inflammatory bowel disease (IBD), colitis, peripheral blood disease, respiratory disease, rheumatic disease, prostate gland enlargement, uterus prolapse, vascular occlusion, kidney stones and hepatic disease. We then excluded 8.933 participants who presented any of the MetS’s components (hypertension, dyslipidemia, high glucose levels and high waist circumference). Since the international harmonized criteria definition states that medical treatment for hypertension, dyslipidemia and high glucose levels should be included within the components’ definitions, we had also excluded participants who regularly take the following pharmaceutics: alpha blockers, beta blockers, calcium channel blockers, Angiotensin receptor blockers, Angiotensin-converting enzyme inhibitors, fibrates, hypoglycemic medications, or insulin. Lastly, we excluded 880 obese participants with elevated BMI. Participants who demonstrated any of these criteria on either the first or second visit were coded as “metabolically impaired” during this visit.

According to this definition of metabolic health, we compared three main groups: metabolically healthy participants on both visits (n = 1131, 16.9%), participants who were healthy on visit 1 but metabolically impaired on visit 2 (n = 985, 12.7%) and participants who were metabolically impaired on both visits (n = 5463, 70.4%).

### Statistical analysis

Categorical variables were presented as numbers and expressed as a percentage of the relevant group. Continuous variables were evaluated for normal distribution and reported as the mean and standard deviation (SD) or as the median and interquartile range (IQR). An estimation of difference in the categorical variables was performed using ${x}^{2}$-test. Continuous variables were assessed using the independent t-test or analysis of variance (ANOVA). In addition, we used a binary logistic regression model to predict the probability of becoming metabolically impaired within 5 years. We adjusted our model for age, gender, non-directly metabolic biomarkers, and baseline metabolic variables, including blood pressure, waist circumference, BMI, HDL-C, FPG, triglycerides, LDL-C and logarithmically transformed hs-CRP. Standardization (z-score) was applied to all variables to enable meaningful comparisons on a common scale, ensuring equitable contributions from each predictor in the analysis, and to accommodate the non-normal distribution of certain variables. All analyses were considered significant at p < 0.05 (two tailed) and were conducted using the SPSS 27.0 statistical package. Graphic analyses of intersections between metabolic components with a known distribution was performed using the Eulerr tool and R package [20].

## Results

The study population consisted of 20,507 apparently healthy participants of which 13,019 were men and 7488 women with a mean age of 44.9 (± 11) years. Participants ranged in age from 18 to 86 years, and 7759 (37.8%) returned for a follow-up visit within 4.8 (± 0.6) years. We compared the entire cohort with the returnees on their first visit (Additional file 1: Table S1). While some variables showed significance, the differences are likely influenced by large sample sizes or other factors. Overall, the cohorts appear similar, representing the same population.

First, we sought to determine the prevalence of individuals without any metabolic abnormality or existing co-morbidities at baseline. Only 5379 participants (26.2% of the cohort) met our exclusion criteria and were defined as “metabolically healthy” on their first visit (Fig. 1). A general comparison of “metabolically healthy” and metabolically impaired participants on the baseline visit is presented in Additional file 2: Table S2. Clinical characteristics by groups of metabolic health status on both visits are presented in Table 1. A comparison of the metabolically healthy and metabolically impaired groups revealed several significant differences: Metabolically healthy individuals were younger, included more women, were less likely to smoke and had lower levels of hs-CRP, fibrinogen, BUN, WBC, RBC, creatinine, hemoglobin, uric acid, liver enzymes, LDH and LDL-C.

**Table 1:** Comparison of the four metabolic groups according to data from the 1st visit

	Healthy on both visits	Healthy on visit 1; unhealthy on visit 2	p-value	Metabolically unhealthy on both visits	p-value (all three groups)	Total population
N (%)	1311 (16.9)	985 (12.7)		5463 (70.4)		7759 (100)
Age, mean (SD)	39.3 (9.7)	42.0 (9.9)	**< 0.001**	48.0 (10.3)	**< 0.001**	45.7 (10.7)
Gender (males) N (%)	740 (56.4)	644 (65.4)	**< 0.001**	3850 (70.5)	**< 0.001**	5234 (67.5)
Current Smoking N (%)	169 (14.2)	119 (13.3)	0.584	573 (11.8)	**0.048**	861 (12.4)
Previous smoker N (%)	197 (15.0)	170 (17.3)	0.136	1230 (22.5)	**< 0.001**	1597 (20.6)
Diastolic, mmHg	71.4 (5.8)	72.5 (5.6)	**< 0.001**	78.6 (8.2)	**< 0.001**	76.6 (8.2)
Systolic, mmHg	111.4 (8.9)	113.1 (8.8)	**< 0.001**	125.5 (15.0)	**< 0.001**	121.6 (14.8)
BMI, kg/${m}^{2}$	23.5 [21.7–25.2]	22.8 [24.4–26.2]	**< 0.001**	26.9 [24.6–29.7]	**< 0.001**	26.3 [23.6–28.5]
Waist circumference, cm	82.0 [75.0–89.0]	85.0 [79.0–92.0]	**< 0.001**	94.0 [87.0–102.0]	**0.000**	91.0 [82.0–99.0]
Average of weekly hours of physical exercise	2.0 [0.0–3.3]	2.0 [0.0–3.7]	0.659	1.7 [0.0–3.0]	**0.008**	2.0 [0.0–3.0]
FPG, mg/dL	86.0 [81.0–90.0]	88.0 [83.0–92.0]	**< 0.001**	92.0 [86.0–100.0]	**< 0.001**	90.0 [85.0–97.0]
HbA1C (%)	5.1 [4.9–5.3]	5.2 [5.0–5.4]	**< 0.001**	5.4 [5.1–5.7]	**< 0.001**	5.3 [5.1–5.6]
Triglycerides mg/dL	74.0 [57.0–97.3]	80.0 [61.0–107.0]	**< 0.001**	116.0 [80.0–163.0]	**< 0.001**	101.0 [72.0–143.0]
HDL mg/dL	61.6 (13.9)	58.5 (13.5)	**< 0.001**	52.7 (13.2)	**< 0.001**	54.9 (13.8)
Hs-CRP, mg/L	0.9 [0.5–1.8]	1.1 [0.6–2.1]	**0.014**	1.6 [0.8–3.5]	**< 0.001**	1.4 [0.7–3.0]
High Hs-CRP > 3.0 md/dL, N (%)	186 (14.4)	144 (15.7)	0.404	1597 (29.5)	** < 0.001**	1927 (25.3)
Creatinine mg/dL	1.0 (0.1)	1.1 (0.1)	**< 0.001**	1.1 (0.2)	**< 0.001**	1.1 (0.2)
High creatinine (males > 1.3 females > 1.1) N (%)	73 (5.9)	68 (7.6)	0.110	513 (9.6)	**< 0.001**	654 (8.8)
Neutrophils, %	58.3 (7.8)	58.4 (7.7)	0.782	59.5 (7.6)	**< 0.001**	59.1 (7.6)
Lymphocytes, %	31.0 (6.8)	30.8 (6.9)	0.581	29.8 (6.8)	**< 0.001**	30.1 (6.8)
Monocytes, %	7.4 [6.3–8.7]	7.5 [6.4–8.7]	0.793	7.4 [6.3–8.6]	0.644	7.4 [6.3–8.6]
Eosinophils, %	2.2 [1.4–3.3]	2.2 [1.4–3.6]	0.284	2.3 [1.5–3.5]	0.384	2.3 [1.4–3.5]
Basophils, %	0.5 [0.4–0.6]	0.5 [0.4–0.6]	0.841	0.5 [0.4–0.6]	0.147	0.5 [0.4–0.6]
fibrinogen, g/L	277.2 (56.5)	284.4 (56.4)	**0.004**	299.6 (60.4)	**< 0.001**	293.9 (60.0)
Albumin, g/L	45.1 (2.5)	45.1 (2.4)	0.962	45.1 (2.3)	0.842	45.1 (2.4)
BUN, mg/dL	13.0 [11.0–16.0]	14.0 [12.0–16.0]	**< 0.001**	14.0 [12.0–17.0]	**< 0.001**	14.0 [12.0–17.0]
PLT, ${x10}^{3}$/µL	242.1 (54.7)	246.1 (52.5)	0.089	250.8 (60.2)	**< 0.001**	248.8 (58.5)
RBC, $x{10}^{6}$/µL	4.7 (0.5)	4.7 (0.4)	**0.001**	4.8 (0.4)	**< 0.001**	4.8 (0.4)
WBC, ${x10}^{3}$/µL	6.3 (1.4)	6.5 (1.4)	**0.001**	6.8 (1.7)	**< 0.001**	6.7 (1.6)
Hemoglobin, g/dL	14.1 (1.3)	14.2 (1.3)	**0.010**	14.4 (1.3)	**< 0.001**	14.3 (1.3)
Bilirubin, mg/dL	0.8 (0.4)	0.8 (0.4)	0.482	0.8 (0.3)	**< 0.001**	0.8 (0.4)
AST U/L	21.0 [18.0–25.0]	22.0 [19.0–25.0]	0.720	23.0 [20.0–26.0]	**< 0.001**	22.0 [19.0–26.0]
ALT U/L	21.6 (10.8)	22.4 (10.2)	0.067	27.1 (13.8)	**< 0.001**	25.6 (13.2)
Uric acid mg/dL	5.0 (1.2)	5.2 (1.2)	**< 0.001**	5.7 (1.3)	**< 0.001**	5.5 (1.3)
Globulin, g/L	28.2 (3.2)	28.4 (3.2)	0.105	28.6 (3.3)	**< 0.001**	28.5 (3.3)
ALP U/L	52.0 [43.0–64.0]	54.0 [46.0–66.0]	**< 0.001**	59.0 [49.0–71.0]	**< 0.001**	57.0 [47.0–69.0]
LDH U/L	297.0 (49.1)	302.3 (49.9)	**0.014**	314.5 (52.9)	**< 0.001**	310.1 (52.4)
GGT U/L	11.0 [7.0–16.0]	13.0 [9.0–18.0]	**0.009**	16.0 [11.0–24.0]	**< 0.001**	15.0 [10.0–22.0]
Phosphorus, mg/dL	3.4 (0.5)	3.4 (0.4)	0.088	3.3 (0.5)	**< 0.001**	3.3 (0.5)
Protein total, g/L	73.3 (4.0)	73.5 (4.0)	0.204	73.6 (3.9)	**0.021**	73.6 (4.0)
Total cholesterol mg/dL	186.5 (32.5)	190.9 (33.5)	**0.002**	198.2 (36.6)	**< 0.001**	195.3 (35.9)
LDL mg/dL	109.4 (28.7)	115.3 (29.6)	**< 0.001**	119.2 (30.9)	**< 0.001**	117.1 (30.6)
High total cholesterol > 200 N (%)	394 (31.5)	334 (37.3)	**0.005**	2457 (46.2)	**< 0.001**	3185 (42.7)
High LDL > 130 N (%)	288 (23.1)	274 (30.6)	**< 0.001**	1850 (35.0)	**< 0.001**	2412 (32.5)
Chloride, mmol/L	104.0 (2.3)	104.0 (2.3)	0.981	103.8 (2.5)	**0.004**	103.9 (2.4)
Potassium, mmol/L	4.3 (0.4)	4.3 (0.4)	**0.045**	4.4 (0.4)	** < 0.001**	4.4 (0.4)
Calcium, mg/dL	9.2 (0.4)	9.3 (0.4)	0.756	9.3 (0.4)	0.569	9.3 (0.4)
Sodium, mmol/L	141.1 (2.5)	141.2 (2.7)	0.138	141.3 (2.6)	**0.030**	141.3 (2.6)
FVC	103.0 [95.0–112.0]	102.0 [94.0–111.0]	0.592	100.0 [92.0–109.0]	**< 0.001**	101.0 [93.0–110.0]
FEV	100.9 (12.8)	100.6 (12.6)	0.564	98.5 (14.8)	**< 0.001**	99.2 (14.2)
FEV/FVC	102.0 [97.0–106.0]	102.0 [97.0–106.0]	0.578	102.0 [97.0–107.0]	0.778	102.0 [97.0–107.0]
Microalbumin urine	3.7 [0.7–8.0]	3.3 [0.9–8.0]	0.452	4.5 [1.1–10.3]	**< 0.001**	4.1 [1.0–9.6]
PHQ	10.0 [9.0–12.0]	10.0 [9.0–12.0]	0.609	10.0 [9.0–12.0]	0.106	10.0 [9.0–12.0]

Values are presented as mean (SD), or median [IQR] for irregular distributed parameters. Bold values are significant.

### Distribution of metabolic syndrome criteria and their development over time

**Fig. 2:**
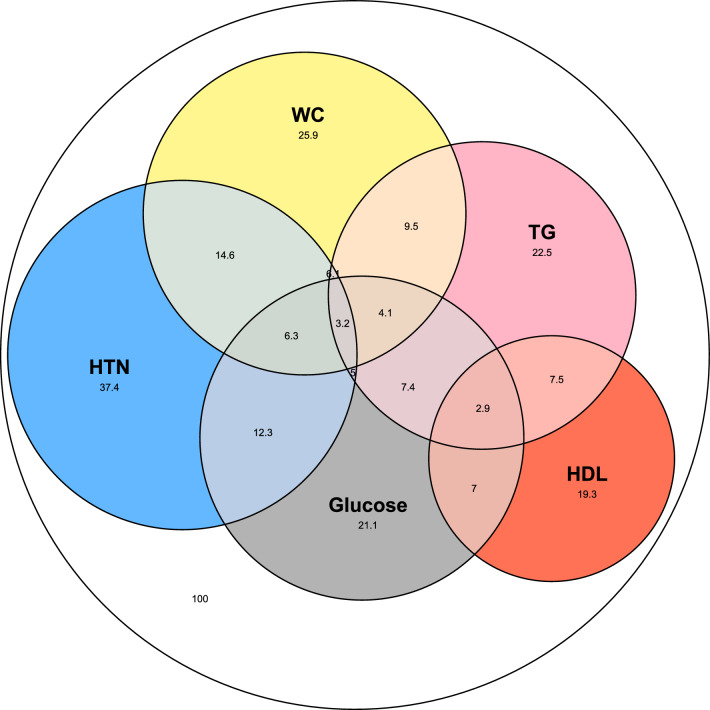
Intersections of metabolic syndrome components and elevated C-reactive protein of the population on visit 1. Euler diagrams of metabolic components’ distribution in the entire cohort on the baseline visit. The white circle represents the entire cohort (20,507, 100%). Each colored circle represents the adjusted prevalence of a metabolic component in the population as measured on the first visit. Grey- elevated glucose levels, blue- hypertension, pink- elevated triglycerides, yellow- high waist circumference and red- low HDL-C. Diagram error: 0.0676, stress: 0.0333

Next, we analyzed the prevalence and distribution of all five metabolic syndrome criteria in our cohort. We found 13,387 individuals (65.3%) that fulfilled at least one metabolic component during their baseline visit (Fig. 2, Additional file 3: Table S3). Hypertension was the most prevalent component (37.4%) followed by WC (25.9%), elevated triglycerides (22.5%), elevated glucose (21.1%); HDL-C was the least prevalent metabolic component (19.3%). Notably, a substantial overlap exists among components, with the highest prevalence seen in the combination of hypertension and elevated WC (14.6%). The most prevalent combination defining a metabolic syndrome, consists of hypertension, WC, and elevated glucose (6.3%), closely followed by hypertension, WC, and elevated triglycerides (6.1%). The presence of all five metabolic criteria simultaneously was observed in 1.3% of the cohort.

**Fig. 3:**
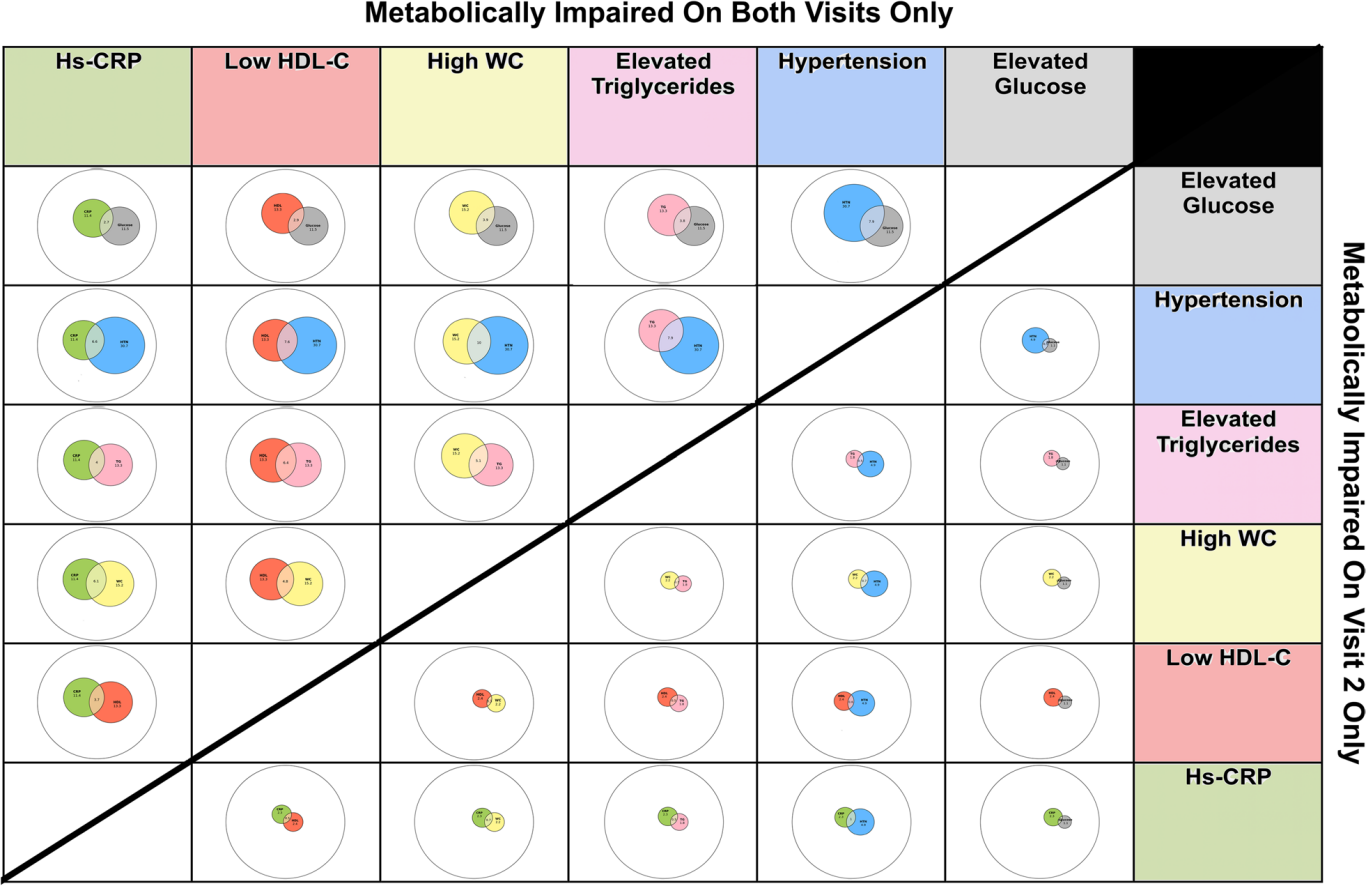
Intersections of single metabolic components and elevated hs-CRP in consistent metabolic impairment and transition to metabolic impairment. Distribution of single metabolic components and hs-CRP on the 2nd visit as Euler diagrams in the consistent metabolically impaired group and the group that became metabolically impaired after a 4.8-year follow-up. The white circle/ellipse on both groups' diagrams represents the entire follow-up population (7759, 100%). Each colored circle/ellipse represents the adjusted prevalence of a metabolic component or CRP of the relevant group respectively as measured on the second visit. Grey- elevated glucose levels, blue- hypertension, pink- elevated triglycerides, yellow- high waist circumference, red- low HDL-C, and green- elevated CRP. The upper left section describes the group that was consistently metabolically impaired on both visits, while the lower right section describes the group that became metabolically impaired on the second visit. Diagram error values: 0.00

We then investigated the relative prevalence of metabolic components across the two measurements. We analyzed the adjusted distribution of metabolic components in the second visit in the following sub-groups: metabolically unhealthy individuals on both visits (5463, 70.4%) and new cases of metabolic impairment on the second visit (985, 12.7%). Each component's intersection with any of the other metabolic components was singled out as presented in Fig. 3 and Additional file 4: Table S4. In an overall observation every component’s relative prevalence was significantly smaller in the group that became metabolically unhealthy over time. The hypertension component was the most prevalent component in both the consistent metabolically impaired and the group that became impaired over time (43.6%, 38.7% of the groups respectively). The least relatively prevalent component on both groups was elevated glucose (16.3% of the consistent metabolically impaired group and 8.9% of the group that became impaired over time).

When examining the overlap of metabolic criteria, it is apparent that the combination of elevated glucose and high waist circumference was approximately 8 folds more prevalent within the consistent metabolically impaired group than individuals who became metabolically impaired over time (5.5% vs 0.7%, p < 0.001, Fig. 3 and Additional file 4: Table S4).

Further analysis of the metabolic components’ combinations revealed a similar appearance (Additional file 4: Table S4). The metabolically consistent impaired group demonstrated a higher relative prevalence of individuals suffering from the combination of three, four or all five components at the same time (combination of all 5 components was 0.8% of the consistent unhealthy group and 0.01% of the group that became unhealthy).

### Active inflammation in metabolically impaired individuals

Since inflammation plays a pivotal role in metabolic abnormalities [21, 22], we additionally assessed elevated levels of hs-CRP (> 3 mg/dL) as indicators of an active inflammatory process. Altogether, 5,505 participants (26.8%) fulfilled this criterion, meaning that a state of active inflammation was more prevalent than all metabolic components, other than hypertension. Interestingly, the combination of hypertension and elevated hs-CRP was the most prevalent among the group that became metabolically impaired over time, while the combination of hypertension and high waist circumference was the most prevalent combination on the baseline visit and on the second visit in the consistent metabolically impaired group.

### Non-metabolic syndrome biomarkers and metabolic health

**Fig. 4:**
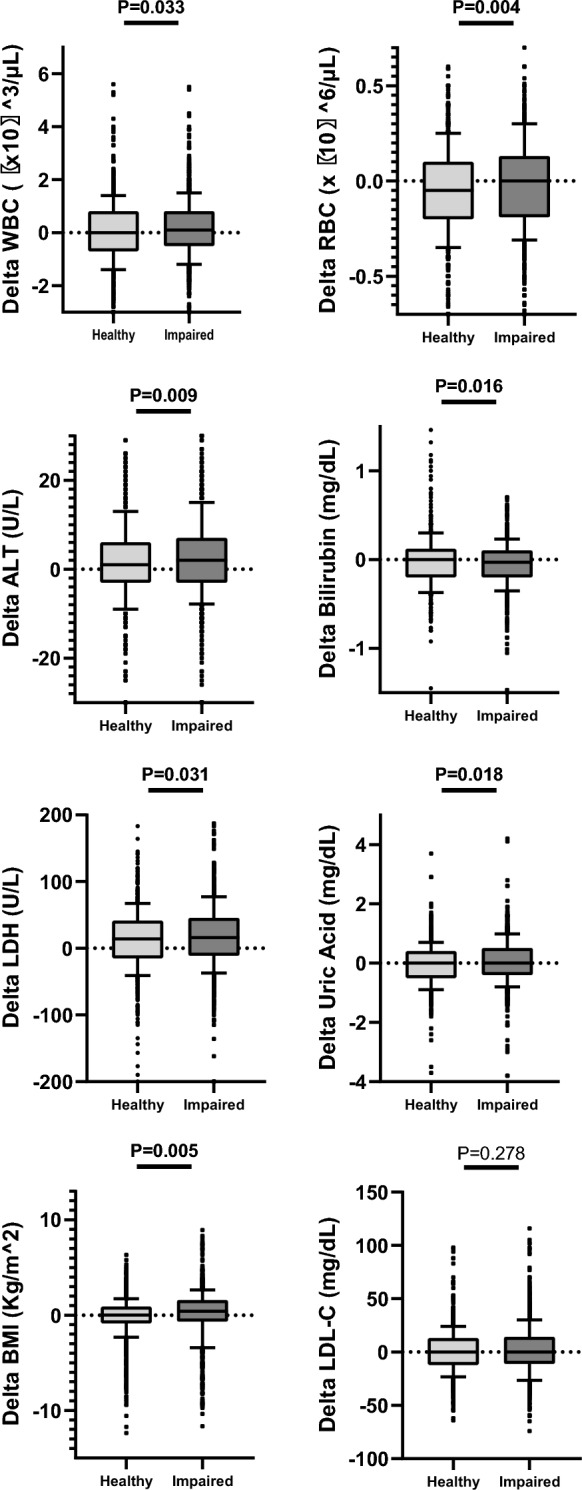
The change in biochemical markers in a 5-year follow-up in consistent metabolic health and transition to metabolic impairment. A comparison between the metabolically healthy group on both visits and the group that became metabolically impaired on the 2nd visit. Box plots represent the distribution of the biomarkers’ deltas between the 10 and 90th percentiles. T-test P-values are presented

Our findings led us to the conclusion that there is a “borderline” state in the development of metabolic impairment. Delving into this metabolic transition, we further compared multiple biomarkers that are not directly connected to the metabolic syndrome, between participants who became metabolically impaired and participants who remained healthy over time. We examined the change between both visits in several biomarkers such as liver enzymes, blood count, electrolytes and lipids (Fig. 4 and Additional file 5: Table S5). The group that became metabolically impaired over time demonstrated a significant elevation in concentrations of WBC, red blood cells (RBC), lactate dehydrogenase (LDH), alanine transaminase (ALT), bilirubin, uric acid and ALP compared to the consistent metabolically healthy group. Surprisingly, the difference in measurements of LDL-C and hs-CRP was not significantly different between the groups. Accordingly, we identified a new group of participants who initially exhibited metabolic health but later developed at least 2 metabolic abnormalities over time. We then conducted a comparison of several 1st visit biomarkers and metabolic measurements in this group with participants who remained healthy over time. Our findings revealed that those who experienced metabolic deterioration had significantly higher levels of BMI, triglycerides, WBC, ALT, ALP, gamma-glutamyl transferase (GGT), fibrinogen, and uric acid, along with significantly lower levels of HDL-C (Table 2). It is noteworthy that this group did exhibit significant higher levels of LDL-C and hs-CRP.

**Table 2:** Comparison between the consistent metabolically healthy group and the group that developed at least 2 metabolic abnormalities according to data from the 1st visit

	Healthy on both visits	Healthy on 1st visit, ≥ 2 metabolic components on 2nd visits	p-value
N	1311	200	
Metabolic syndrome N (%)	0 (0.0)	39 (19.5)	
Age, mean (SD)	39.3 (9.7)	43.1 (9.8)	**< 0.001**
Gender (males) N (%)	740 (56.4)	135 (67.5)	**0.003**
Diastolic, mmHg	71.4 (5.8)	72.7 (5.9)	**0.006**
Systolic, mmHg	111.4 (8.9)	113.8 (9.1)	**< 0.001**
BMI, kg/${m}^{2}$	23.5 [21.7–25.2]	25.9 [24.2–27.7]	**< 0.001**
Waist circumference, cm	82.0 [75.0–89.0]	88.0 [82.0–95.0]	**< 0.001**
FPG, mg/dL	86.0 [81.0–90.0]	88.0 [83.0–92.0]	**0.001**
HbA1C (%)	5.1 [4.9–5.3]	5.2 [4.9–5.4]	0.176
Triglycerides mg/dL	74.0 [57.0–97.3]	93.0 [69.5–121.5]	**< 0.001**
HDL mg/dL	61.6 (13.9)	56.1 (13.0)	**< 0.001**
Hs-CRP, mg/L	0.9 [0.5–1.8]	1.3 [0.8–2.5]	**0.032**
High Hs-CRP > 3.0 md/dL, N (%)	186 (14.4)	34 (20.0)	0.056
Creatinine mg/dL	1.0 (0.1)	1.1 (0.1)	**0.010**
Neutrophils, %	58.3 (7.8)	58.9 (7.3)	0.337
Lymphocytes, %	31.0 (6.8)	30.5 (6.8)	0.370
Monocytes, %	7.4 [6.3–8.7]	7.5 [6.3–8.6]	0.940
Eosinophils, %	2.2 [1.4–3.3]	2.2 [1.3–3.5]	0.426
Basophils, %	0.5 [0.4–0.6]	0.5 [0.4–0.6]	0.956
fibrinogen, g/L	277.2 (56.5)	293.5 (64.4)	**< 0.001**
Albumin, g/L	45.1 (2.5)	44.8 (2.4)	0.194
BUN, mg/dL	13.0 [11.0–16.0]	14.0 [11.5–16.0]	0.713
PLT, ${x10}^{3}$/µL	242.1 (54.7)	240.7 (52.9)	0.742
RBC, $x{10}^{6}$/µL	4.7 (0.5)	4.8 (0.4)	**0.045**
WBC, ${x10}^{3}$/µL	6.2 [5.3–7.1]	6.5 [5.6–7.6]	**< 0.001**
Hemoglobin, g/dL	14.1 (1.3)	14.3 (1.3)	**0.016**
Bilirubin, mg/dL	0.7 [0.6–1.0]	0.7 [0.6–0.9]	0.263
AST U/L	21.0 [18.0–25.0]	22.0 [19.0–25.0]	0.359
ALT U/L	19.0 [15.0–25.0]	22.0 [17.0–29.0]	**0.008**
Uric acid mg/dL	5.0 (1.2)	5.4 (1.3)	**< 0.001**
Globulin, g/L	28.2 (3.2)	28.5 (3.0)	0.240
ALP U/L	52.0 [43.0–64.0]	55.0 [46.0–66.0]	**0.033**
LDH U/L	294.0 [264.0–326.0]	301.0 [272.5–330.5]	0.131
GGT U/L	11.0 [7.0–16.0]	14.0 [10.0–19.0]	**0.029**
Phosphorus, mg/dL	3.4 (0.5)	3.4 (0.4)	0.082
Protein total, g/L	73.3 (4.0)	73.3 (3.7)	0.908
Total cholesterol mg/dL	186.5 (32.5)	194.1 (32.7)	**0.005**
LDL mg/dL	109.4 (28.7)	119.0 (29.6)	**< 0.001**
Chloride, mmol/L	104.0 (2.3)	104.0 (2.1)	0.967
Potassium, mmol/L	4.3 (0.4)	4.3 (0.3)	0.693
Calcium, mg/dL	9.2 (0.4)	9.2 (0.4)	0.532
Sodium, mmol/L	141.1 (2.5)	141.2 (2.8)	0.524

Values are presented as mean (SD), or median [IQR] for irregular distributed parameters. Bold values are significant.

**Fig. 5:**
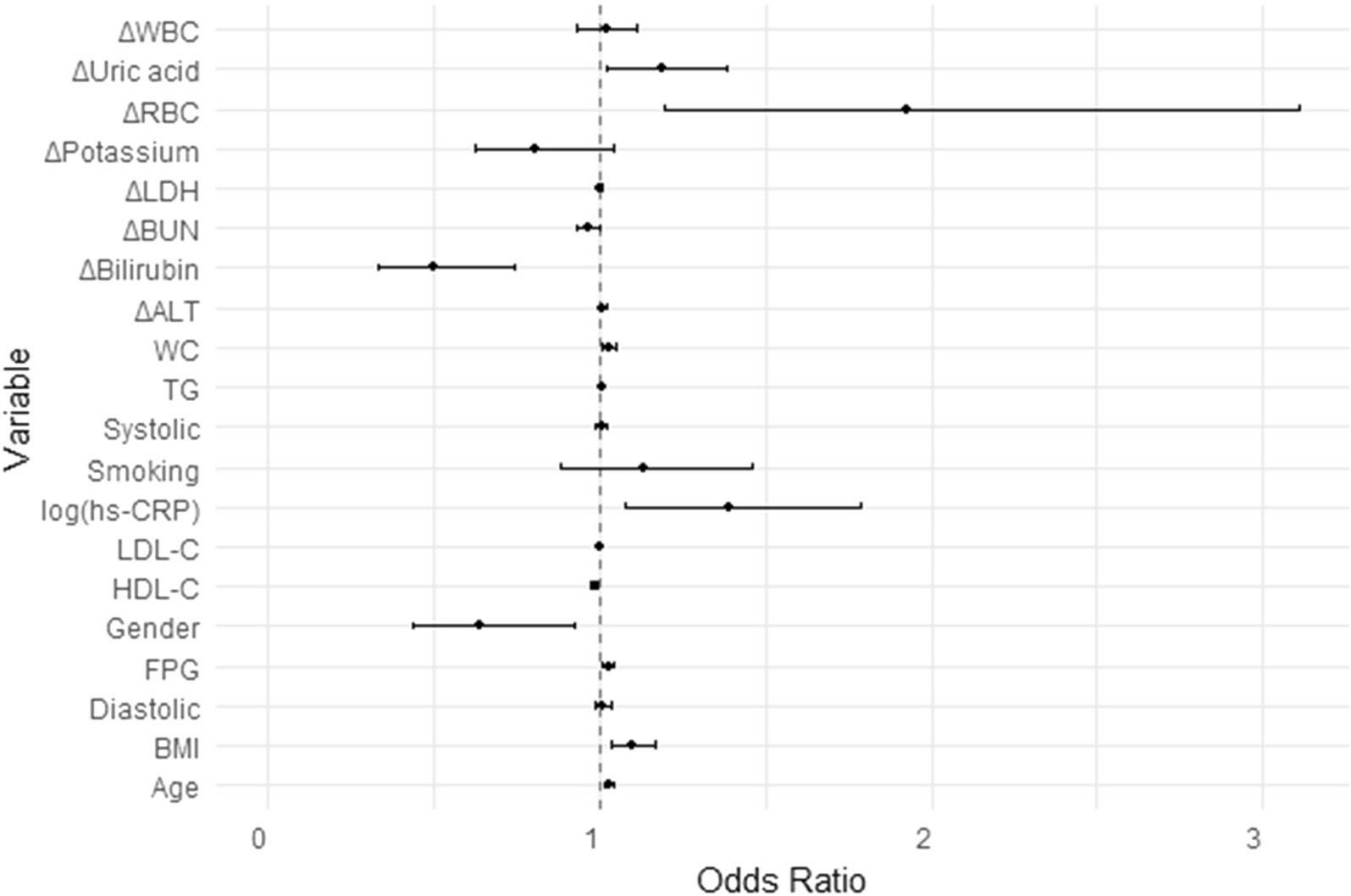
Odds-ratio plot for predicting metabolic impairment risk within 5 years

Finally, we conducted a logistic regression analysis to predict a transition to metabolic impairment within 5 years (Table 3 and Fig. 5. Univariate models are presented in Additional file 6: Table S6). The analysis revealed significant contributions of ΔRBC (OR = 1.9, p = 0.007), gender (OR = 0.6, p = 0.018), ΔBilirubin (OR = 0.5, p < 0.001), ΔUric acid (OR = 1.2, p = 0.025), BMI (OR = 1.1, p = 0.003), and log(hs-CRP) (OR = 1.4, p = 0.011). Additional variables like, LDH, FPG, WC, triglycerides, HDL-C and age were significant with a lower contribution to the regression model.

**Table 3:** Covariate regression model for the probability of becoming metabolically impaired within 5 years*

Variable	p-value	OR	CI
Gender (males)	**0.018**	**0.639**	0.441–0.927
Age (first visit) years	**< 0.001**	**1.031**	1.017–1.045
Deltas of non-metabolic biomarkers			
Delta RBC	**0.007**	**1.928**	1.196–3.108
Delta bilirubin	**< 0.001**	**0.498**	0.333–0.744
Delta LDH	**0.028**	**1.003**	1.000–1.005
Delta uric acid	**0.025**	**1.191**	1.022–1.387
Metabolic values from the 1st visit			
Diastolic	0.458	1.010	0.984–1.036
Systolic	0.403	1.007	0.990–1.025
Waist circumference	**0.012**	**1.029**	1.006–1.051
BMI	**0.003**	**1.099**	1.033–1.169
FPG	**0.002**	**1.027**	1.010–1.045
Triglycerides	**0.023**	**1.005**	1.001–1.009
HDL-C	**0.003**	**0.985**	0.975–0.995
LDL-C	0.678	0.999	0.995–1.003
Log (Hs-CRP)	**0.011**	**1.390**	1.080–1.790

^*^All predictor variables were standardized before fitting the regression model. The model includes all coefficients listed in this table (bold values are significant) and the following non-significant coefficients: currently/previously smoking, delta WBC, delta BUN, delta potassium, delta ALT, delta creatinine. The sample comprised 1505 participants, with 817 individuals transitioning to metabolic impairment over time

## Discussion

We report here the relatively low prevalence of apparently healthy individuals without any metabolic abnormality (roughly 26% of our cohort). The most prevalent metabolic syndrome criterion in our cohort was hypertension. Furthermore, we found several small significant changes in non-direct-metabolic biomarkers in participants that changed their status of metabolic health over time.

The groups of our study, especially the group that developed metabolic abnormalities over time, provide an opportunity to evaluate the metabolic health transition point, along with continuous metabolic health or impairment statuses. Our findings imply the possible existence of antecedent biomarkers for metabolic impairment. Equally important, our results may also point out the importance of aggressive early screening for hypertension as an indicator of early metabolic impairment and associated co-morbidities [23, 24], especially if accompanied with current active inflammation.

The significant prevalence of hypertension in comparison to all other metabolic components could be explained by the influence of the cohort's mean age and males as the prevalent gender. These results are in line with previous studies that found hypertension to be the most prevalent component among younger men and older men and women [25]. However, it is important to mention that some studies in different populations found the low HDL-C component as the most prevalent component, followed by hypertension [26, 27]. Nevertheless, since blood pressure (especially systolic blood pressure) tends to increase with age [28], and previous studies have shown that its prevalence is significantly high and somewhat stable over time [29], our findings further support the need to aggressively screen for elevated blood pressure in patients with any metabolic abnormality.

Previous studies suggest a strong association between MetS and subclinical inflammation demonstrated in elevated levels of CRP [16]. Thus, it is not surprising that our results present increased prevalence of active inflammation among metabolically impaired individuals. The relatively large intersection of hypertension and elevated hs-CRP on the group that became metabolically impaired overtime, could suggest that the emergence of hypertension at the metabolic transition process is widely accompanied by active inflammation. Previous studies like the Women’s Health Study and the Framingham Offspring Study strengthen this concept by showing that CRP could independently predict the development of new-onset hypertension [30, 31].

Metabolic health has a time-varying nature. For this reason, the small changes we found in non-direct-metabolic biomarkers may imply subjects at risk of developing metabolic syndrome or diseases. Amongst these findings, a relative elevation of WBC in participants that became metabolically impaired could be explained by its positive association to hyperglycemia, low HDL-C and hypertriglyceridemia [32]. Another interesting finding is the relatively lower decrease and even elevation of RBC count in the metabolically impaired group. We previously found that enhanced erythropoiesis is associated with the multiplicity of MetS components [33], while other studies indicate that insulin resistance mechanisms support erythropoiesis [34, 35], these findings collectively point to a potential explanation for our observation. The RBC change’s clinical relevance is attributed to the fact that an alteration of erythrocytes count, shape or elasticity could contribute to vascular damage and reduced blood flow, as the cells’ aggregative and adhesive qualities change [36]. Sub-sequentially this may lead to a decrease of circulating oxygen, insulin and glucose which could catalyze the progression of metabolic abnormalities like diabetes [37]. Thus, it is not surprising that elevated RBC count could contribute to transition in metabolic health and may potentially serve as an early indicator for metabolic deterioration.

Other findings like the elevated LDH could derive from an extensive tissue damage that causes its release to the bloodstream due to hypertension or diabetes emergence [38]. Bilirubin was previously found to be negatively correlated with MetS 39], while liver enzymes such as ALT seem to be higher in metabolic abnormalities [40].

Surprisingly, there was no significant difference of the change in concentrations of LDL-C in the group that became metabolically impaired. Even though LDL-C was found in numerous studies to be an independent risk factor of CVD [41], it is not part of the official MetS criteria. The exact association between LDL-C and the MetS seems to be controversial. Some studies report LDL-C has no significant correlation with MetS prevalence [42, 43], while others claim that LDL-C levels are not only associated with the MetS, but can also serve as a predictor for the syndrome's development [44]. In addition, it is not clear what are the levels of LDL-C concentration responsible for an increase of MetS prevalence in the overall population [45]. A possible cause for our finding could be that a change in LDL-C concentrations depends on the change in LDL-C subclasses. While small density LDL-C particles seem to increase in the MetS, there is a significant reduction of large LDL-C particles. This elevation and decrease could affect the LDL particle number but not result in a change of the total LDL-C concentration [46, 47]. Another possible speculation is that LDL-C concentration simply does not change rapidly on the verge of change of metabolic status. Furthermore, it is worth noting that 56 participants (5.7%) in the group that developed metabolic impairment initiated a new lipid-lowering treatment between visits, which may have influenced the results.

Unfortunately, this study reflects the unfavorable reality of metabolic health and illness. Even though this study's population consisted of apparently healthy, work-aged participants, our analyses show significant high rates of various illnesses among them, mainly metabolic abnormalities. To the best of our knowledge, numerous studies have discussed the definitions of metabolic abnormalities, but few have mentioned an agreeable definition of health, other than the MHO definition. Nevertheless, even when considering MHO as an accepted health definition it is important to remember that metabolic health is a transient status in nature [48], along with the very fact that it is yet to be clear what are the best antecedent biomarkers for MHO individuals who may progress to impaired cardiometabolic health.

Therefore, we believe there is a crucial value of evaluating and accurately defining metabolic health, in order to investigate borderline conditions, preventable medical cases that could be invertible using aggressive screening of metabolic or other indicators.

The main limitation of this study is that our cohort is comprised of participants in a health screening program, thus it is not a population-based sample. Additionally, our cohort experiences loss of follow-up, which could potentially cause some selection bias regarding the results. However, our cohort primarily included working participants whose periodic attendance is facilitated by workplace benefits, contributing to a high rate of participation (91.6% of those asked). Also, comparing both cohorts revealed non-fundamental differences. It is important to mention that although participants are invited to our center several months in advance, it is possible that some may have chosen to attend due to a recent illness, which could have served as a motivating factor. To address this, we took specific measures for participants who exhibited increased CRP values (hs-CRP > 10 mg/dL). We reinvited them for a second CRP examination, and only the values from the second blood test were included in our analysis. In addition, while the observed differences in biomarkers are statistically significant, they exhibit relatively modest magnitudes. Consequently, their clinical significance is limited, possibly due to the dichotomization around cut-off values. The group that developed metabolic impairment was already close to impairment levels initially, suggesting this outcome could be an artifact of the chosen cut-offs. Further research is essential to determine precise cut-off points and personalize them for more reliable and relevant studies.

## Conclusions

This study provides further evidence that metabolic abnormality is very common and should be taken into consideration in terms of early screening and treatment. Hypertension, especially when combined with active inflammation, is a prime candidate for agxgressive screening of patients in increased risk to develop MetS over time. Further investigation is needed to examine the potentially predicting role of additional biomarkers like blood count, liver enzymes, LDH and uric acid. We recommend further research focused on the transition between metabolic health and abnormality to improve detection abilities and reduce future morbidity.

### Supplementary Information

**Additional file 1: Table S1.** Demographic and metabolic comparison of the entire population and the population of returnees.

**Additional file 2: Table S2.** Comparison between metabolically healthy and impaired participants on visit 1. Values are presented as mean (SD), or median [IQR] for irregular distributed parameters.

**Additional file 3: Table S3.** Prevalence of metabolic components and elevated hs-CRP in the entire cohort

**Additional file 4: Table S4.** Comparison of metabolic components and elevated hs-CRP in consistent metabolic impairment and transition to metabolic impairment. Comparison between the group that became metabolically impaired and the group that was metabolically impaired on both visits as measured on the 2nd visit.

**Additional file 5: Table S5.** Deltas of biomarkers presented as mean and IQR in metabolic health and transition to metabolic impairment. Comparison of the group that remained healthy on both visits and the group that became metabolically impaired as measured on the 2nd visit.

**Additional file 6: Table S6.** Univariate regression for metabolic impairment. The model includes delta values of biomarkers alongside metabolic and demographic components from the first visit.

## Data Availability

The data that support the findings of this study are available from the corresponding author upon reasonable request.

## Abbreviations

TAMCIS: Tel Aviv Medical Center Inflammation Survey
hs-CRP: High sensitivity C-reactive protein
WBC: White blood cells
RBC: Red blood cells
LDL-C: Low density lipoprotein cholesterol
MetS: Metabolic syndrome
CVD: Cardiovascular diseases
HDL-C: High density lipoprotein cholesterol
MHO: Metabolically healthy obesity
HemA1C: Glycohemoglobin
BMI: Body mass index
WC: Waist circumference
FPG: Fasting plasma glucose
cm: Centimeter
mmHg: Millimeters of mercury
ADA: American diabetes association
WHO: World health organization
CVA: Cerebro-vascular accident
IBD: Inflammatory bowel disease
SD: Standard deviation
IQR: Interquartile range
ANOVA: Analysis of variance
WBC: White blood cells
ALP: Alkaline phosphatase
GGT: Gamma-glutamyl transferase
ALT: Alanine transaminase
OR: Odds ratio
HTN: Hypertension
PBD: Peripheral blood disease
TG: Triglycerides
PLT: Platelets
LDH: Lactate dehydrogenase
TG: Triglycerides
