# Supplementary material for: Transitioning from having no metabolic abnormality nor obesity to metabolic impairment in a cohort of apparently healthy adults
Source: Cardiovasc Diabetol. 2023 Aug 26;22:226. doi: 10.1186/s12933-023-01954-w (PMC10463945; doi:10.1186/s12933-023-01954-w)
Supplement: Supplementary file 1 — Additional file 1: Table S1. Demographic and metabolic comparison of the entire population and the population of returnees. [file 12933_2023_1954_MOESM1_ESM.docx]

**Table S1: Demographic and metabolic comparison of the entire population and the population of returnees**

|  | **Total population- visit 1** | **Population of returnees- visit 1** | **P-value** |
| --- | --- | --- | --- |
| **N** | 20507 | 7759 |  |
| **Age, mean (SD)** | 44.9 (11.0) | 45.7 (10.7) | **<0.001** |
| **Gender (males) N (%)** | 13019 (63.6) | 5234 (67.5) | **<0.001** |
| **Hypertension N (%)** | 7673 (37.4) | 2725 (35.1) | **<0.001** |
| **High waist circumference N (%)** | 5320 (25.9) | 1946 (25.1) | 0.086 |
| **High glucose N (%)** | 4336 (21.1) | 1427 (18.4) | **<0.001** |
| **High triglycerides N (%)** | 4611 (22.5) | 1708 (22.8) | 0.532 |
| **Low HDL-C N (%)** | 3952 (19.3) | 1138 (14.7) | **<0.001** |
| **BMI, kg/**$\boldsymbol{m}^{\boldsymbol{2}}$**, mean (SD)** | 26.4 (4.3) | 26.3 (4.1) | 0.077 |
| **Metabolic syndrome N (%)** | 3449 (16.8) | 1145 (14.8) | **<0.001** |
