# Supplementary material for: Transitioning from having no metabolic abnormality nor obesity to metabolic impairment in a cohort of apparently healthy adults
Source: Cardiovasc Diabetol. 2023 Aug 26;22:226. doi: 10.1186/s12933-023-01954-w (PMC10463945; doi:10.1186/s12933-023-01954-w)
Supplement: Supplementary file 2 — Additional file 2: Table S2. Comparison between metabolically healthy and impaired participants on visit 1. Values are presented as mean (SD), or median [IQR] for irregular distributed parameters. [file 12933_2023_1954_MOESM2_ESM.docx]

**Table S2: Comparison between metabolically healthy and impaired participants on visit 1.**

Values are presented as mean (SD), or median [IQR] for irregular distributed parameters.

|  | **Metabolically Healthy** | **Metabolically impaired** | **P-value** | **Total population** |
| --- | --- | --- | --- | --- |
| **N (%)** | 5379 (26.2) | 15128 (73.8) |  | 20507 (100) |
| **Age, mean (SD)** | 39.0 (9.7) | 47.0 (10.7) | **0.000** | 44.9 (11.0) |
| **Gender (males) N (%)** | 2984 (55.5) | 10035 (66.5) | **<0.001** | 13019 (63.6) |
| **Metabolic components** | | | | |
| **Current Smoking N (%)** | 895 (16.7) | 2410 (16.1) | 0.338 | 3305 (16.3) |
| **Previous smoker N (%)** | 985 (18.4) | 3934 (26.3) | **<0.001** | 4919 (24.2) |
| **Diastolic, mmHg** | 71.6 (6.0) | 78.7 (9.1) |  | 76.8 (8.9) |
| **Systolic, mmHg** | 111.9 (9.2) | 125.8 (15.9) |  | 122.2 (15.7) |
| **BMI, kg/**$\boldsymbol{m}^{\boldsymbol{2}}$ | 23.2 [21.8-25.5] | 26.8 [24.4-29.8] |  | 25.8 [23.4-28.7] |
| **Waist circumference, cm** | 82.0 [75.0-89.0] | 93.0 [86.0-101.0] |  | 90.0 [82.0-98.0] |
| **Average of weekly hours of physical exercise** | 1.7 [0.0-3.0] | 1.5 [0.0-3.0] | <**0.001** | 1.7 [0.0-3.0] |
| **FPG, mg/dL** | 86.0 [81.0-91.0] | 91.0 [85.0-100.0] |  | 90.0 [84.0-97.0] |
| **HbA1C (%)** | 5.2 [5.0-5.4] | 5.4 [5.2-5.7] |  | 5.4 [5.1-5.6] |
| **Triglycerides mg/dL** | 77.0 [59.0-100.0] | 113.0 [79.0-162.0] |  | 100.0 [71.0-144.0] |
| **HDL mg/dL** | 58.2 [50.3-68.6] | 50.0 [43.2-59.2] |  | 52.0 [44.8-62.1] |
| **Biochemical components** | | | | |
| **Hs-CRP, mg/L** | 0.9 [0.5-2.0] | 1.7 [0.8-3.7] | <**0.001** | 1.4 [0.7-3.2] |
| **High Hs-CRP >3.0 mg/dL, N (%)** | 843 (15.8) | 4662 (31.4) | **<0.001** | 5505 (27.3) |
| **Creatinine mg/dL** | 1.0 (0.2) | 1.1 (0.2) | **<0.001** | 1.1 (0.2) |
| **High creatinine (males >1.3 females >1.1) N (%)** | 296 (5.8) | 1309 (8.9) | **<0.001** | 1605 (8.1) |
| **Neutrophils, %** | 58.3 (8.0) | 59.3 (7.8) | **<0.001** | 59.0 (7.8) |
| **Lymphocytes, %** | 30.9 (7.1) | 29.9 (6.9) | **<0.001** | 30.2 (7.0) |
| **Monocytes, %** | 7.5 (1.9) | 7.6 (1.8) | 0.192 | 7.6 (1.9) |
| **Eosinophils, %** | 2.2 [1.4-3.5] | 2.3 [1.5-3.5] | 0.512 | 2.2 [1.4-3.5] |
| **Basophils, %** | 0.5 [0.4-0.7] | 0.5 [0.4-0.6] | **<0.001** | 0.5 [0.4-0.6] |
| **fibrinogen, g/L** | 273.7 [239.8-312.9] | 298.0 [258.1-337.7] | **<0.001** | 290.4 [253.0-331.0] |
| **Albumin, g/L** | 45.2 (2.6) | 45.2 (2.5) | 0.405 | 45.2 (2.5) |
| **BUN, mg/dL** | 13.0 [11.0-16.0] | 14.0 [12.0-17.0] | **<0.001** | 14.0 [12.0-16.0] |
| **PLT,** ${\boldsymbol{x}\boldsymbol{10}}^{\boldsymbol{3}}$**/µL** | 245.5 (56.3) | 249.6 (59.9) | **<0.001** | 248.5 (59.0) |
| **RBC,** $\boldsymbol{x}\boldsymbol{10}^{\boldsymbol{6}}$**/µL** | 4.7 (0.5) | 4.8 (0.5) | **<0.001** | 4.8 (0.5) |
| **WBC,** ${\boldsymbol{x}\boldsymbol{10}}^{\boldsymbol{3}}$**/µL** | 6.3 [5.4-7.3] | 6.8 [5.8-7.9] | **<0.001** | 6.6 [5.7-7.7] |
| **Hemoglobin, g/dL** | 14.1 (1.3) | 14.4 (1.3) | **<0.001** | 14.3 (1.3) |
| **Bilirubin, mg/dL** | 0.7 [0.6-0.9] | 0.7 [0.5-0.9] | **<0.001** | 0.7 [0.5-0.9] |
| **AST U/L** | 21.0 [19.0-25.0] | 23.0 [19.0-26.0] | **<0.001** | 22.0 [19.0-26.0] |
| **ALT U/L** | 19.0 [15.0-25.0] | 24.0 [18.0-31.0] | **<0.001** | 22.0 [17.0-30.0] |
| **Uric acid mg/dL** | 5.0 (1.2) | 5.6 (1.3) | **<0.001** | 5.5 (1.3) |
| **Globulin, g/L** | 28.4 (3.1) | 28.7 (3.8) | **<0.001** | 28.6 (3.6) |
| **ALP U/L** | 55.0 [45.0-66.0] | 61.0 [50.0-74.0] | **<0.001** | 60.0 [49.0-72.0] |
| **LDH U/L** | 294.0 [267.0-327.0] | 310.0 [279.0-345.0] | **<0.001** | 306.0 [275.0-341.0] |
| **GGT U/L** | 12.0 [9.0-18.0] | 17.0 [12.0-26.0] | **<0.001** | 16.0 [11.0-24.0] |
| **Phosphorus, mg/dL** | 3.5 (0.5) | 3.3 (0.5) | **<0.001** | 3.4 (0.5) |
| **Protein total, g/L** | 73.6 (4.0) | 73.8 (4.0) | **<0.001** | 73.8 (4.0) |
| **Total cholesterol mg/dL** | 188.2 (33.3) | 198.8 (37.8) | **<0.001** | 196.0 (37.0) |
| **LDL mg/dL** | 111.4 (29.8) | 120.3 (31.7) | **<0.001** | 118.0 (31.5) |
| **High LDL >130 N (%)** | 1313 (25.4) | 5370 (36.6) | **< 0.001** | 6683 (33.7) |
| **Electrolytes** | | | | |
| **Chloride, mmol/L** | 103.9 (2.3) | 103.8 (2.5) | **<0.001** | 103.8 (2.5) |
| **Potassium, mmol/L** | 4.3 [4.0-4.5] | 4.3 [4.1-4.6] | **<0.001** | 4.3 [4.1-4.6] |
| **Calcium, mg/dL** | 9.3 (0.4) | 9.3 (0.4) | **<0.001** | 9.3 (0.4) |
| **Sodium, mmol/L** | 140.9 (2.6) | 141.1 (2.9) | **0.002** | 141.0 (2.8) |
| **Urine** | | | | |
| **Microalbumin urine** | 3.6 [0.8-8.4] | 4.3 [1.1-10.1] | **<0.001** | 4.1 [1.0-9.6] |
