# Supplementary material for: Transitioning from having no metabolic abnormality nor obesity to metabolic impairment in a cohort of apparently healthy adults
Source: Cardiovasc Diabetol. 2023 Aug 26;22:226. doi: 10.1186/s12933-023-01954-w (PMC10463945; doi:10.1186/s12933-023-01954-w)
Supplement: Supplementary file 3 — Additional file 3: Table S3. Prevalence of metabolic components and elevated hs-CRP in the entire cohort [file 12933_2023_1954_MOESM3_ESM.docx]

**Table S3: Prevalence of metabolic components and elevated hs-CRP in the entire cohort.**

| **Number of metabolic components** | **Components combination** | **Prevalence, %** | **Prevalence with elevated hs-CRP, %** |
| --- | --- | --- | --- |
| 1 | DM | 21.1 | 7.3 |
|  | HTN | 37.4 | 11.9 |
|  | TG | 22.5 | 8.6 |
|  | WC | 25.9 | 12.1 |
|  | HDL | 19.3 | 6.9 |
|  | Hs-CRP | 26.8 |  |
| 2 | DM+ HTN | 12.3 | 4.7 |
|  | DM+ TG | 7.4 | 3.3 |
|  | DM+ WC | 8.7 | 3.3 |
|  | DM+ HDL | 7.0 | 2.5 |
|  | HTN+ TG | 11.7 | 4.7 |
|  | HTN+ WC | 14.6 | 7.1 |
|  | HTN+ HDL | 8.0 | 3.3 |
|  | TG+ WC | 9.5 | 5.0 |
|  | TG+ HDL | 7.5 | 3.3 |
|  | WC+ HDL | 7.0 | 3.9 |
| 3 | DM+ HTN+ TG | 5.0 | 2.3 |
|  | DM+ HTN+ WC | 6.3 | 3.3 |
|  | DM+ HTN+ HDL | 3.8 | 1.7 |
|  | DM+ TG+ WC | 4.1 | 2.3 |
|  | DM+ TG+ HDL | 2.9 | 1.4 |
|  | DM+ WC+ HDL | 2.8 | 1.6 |
|  | HTN+ TG+ WC | 6.1 | 3.2 |
|  | HTN+ TG+ HDL | 4.0 | 1.9 |
|  | HTN+ WC+ HDL | 3.9 | 2.3 |
|  | TG+ WC+ HDL | 3.6 | 2.1 |
| 4 | DM+ HTN+ TG+ WC | 3.2 | 1.8 |
|  | DM+ HTN+ TG+ HDL | 2.0 | 1.0 |
|  | DM+ HTN+ WC+ HDL | 2.0 | 1.2 |
|  | DM+ TG+ WC+ HDL | 1.7 | 1.1 |
|  | HTN+ TG+ WC+ HDL | 2.3 | 1.4 |
| 5 | DM+ HTN+ TG+ WC+ HDL | 1.3 | 0.8 |
