# Supplementary material for: Transitioning from having no metabolic abnormality nor obesity to metabolic impairment in a cohort of apparently healthy adults
Source: Cardiovasc Diabetol. 2023 Aug 26;22:226. doi: 10.1186/s12933-023-01954-w (PMC10463945; doi:10.1186/s12933-023-01954-w)
Supplement: Supplementary file 4 — Additional file 4: Table S4. Comparison of metabolic components and elevated hs-CRP in consistent metabolic impairment and transition to metabolic impairment. Comparison between the group that became metabolically impaired and the group that was metabolically impaired on both visits as measured on the 2nd visit. [file 12933_2023_1954_MOESM4_ESM.docx]

**Table S4: Comparison of metabolic components and elevated hs-CRP in consistent metabolic impairment and transition to metabolic impairment.**

Comparison between the group that became metabolically impaired and the group that was metabolically impaired on both visits as measured on the 2^nd^ visit.

|  | **Healthy on visit 1; not healthy on  visit 2** | **Not healthy on both visits** | **P-value** |
| --- | --- | --- | --- |
| **Elevated glucose, N (%)** | 88 (8.9) | 892 (16.3) | **<0.001** |
| **HTN, N (%)** | 381 (38.7) | 2384 (43.6) | **<0.001** |
| **Elevated TG, N (%)** | 140 (15.4) | 1032 (27.1) | **<0.001** |
| **High WC, N (%)** | 172 (17.5) | 1182 (21.7) | **<0.001** |
| **Low HDL-C, N (%)** | 190 (19.3) | 1034 (18.9) | **<0.001** |
| **Elevated hs-CRP, N (%)** | 182 (21.1) | 881 (26.3) | **<0.001** |
| **Elevated glucose& HTN, N (%)** | 36 (3.7) | 613 (11.2) | **<0.001** |
| **Elevated glucose& elevated TG, N (%)** | 8 (0.8) | 294 (5.5) | **<0.001** |
| **Elevated glucose& high WC, N (%)** | 7 (0.7) | 302 (5.5) | **<0.001** |
| **Elevated glucose& low HDL-C, N (%)** | 13 (1.3) | 226 (4.1) | **<0.001** |
| **Elevated glucose& elevated hs-CRP, N (%)** | 12 (1.2) | 210 (4.0) | **<0.001** |
| **Hypertension& elevated TG, N (%)** | 38 (3.9) | 613 (12.1) | **<0.001** |
| **HTN& high WC, N (%)** | 58 (5.9) | 777 (14.2) | **<0.001** |
| **HTN& low HDL-C, N (%)** | 47 (4.8) | 591 (10.8) | **<0.001** |
| **HTN& elevated hs-CRP, N (%)** | 80 (8.3) | 509 (10.3) | **<0.001** |
| **Elevated TG& high WC, N (%)** | 22 (2.2) | 393 (7.2) | **<0.001** |
| **Elevated TG& low HDL-C, N (%)** | 38 (4.0) | 499 (9.2) | **<0.001** |
| **Elevated TG& elevated hs-CRP, N (%)** | 35 (3.8) | 309 (8.3) | **<0.001** |
| **High WC& low HDL-C, N (%)** | 24 (2.4) | 374 (6.9) | **<0.001** |
| **High WC& elevated hs-CRP, N (%)** | 55 (5.6) | 476 (8.7) | **<0.001** |
| **Low HDL-C& elevated hs-CRP, N (%)** | 39 (4.0) | 284 (5.3) | **<0.001** |
| **Elevated glucose& HTN& elevated TG, N (%)** | 5 (0.5) | 220 (4.1) | **<0.001** |
| **Elevated glucose& HTN& high WC, N (%)** | 5 (0.5) | 246 (4.5) | **<0.001** |
| **Elevated glucose& HTN& low HDL-C, N (%)** | 17 (1.7) | 207 (3.8) | **<0.001** |
| **Elevated glucose& elevated TG& high WC, N (%)** | 2 (0.2) | 128 (2.3) | **<0.001** |
| **Elevated glucose& elevated TG& low HDL-C, N (%)** | 4 (0.4) | 155 (2.9) | **<0.001** |
| **Elevated glucose& high WC& low HDL-C, N (%)** | 2 (0.2) | 115 (2.1) | **<0.001** |
| **HTN& elevated TG& high WC, N (%)** | 11 (1.1) | 277 (5.1) | **<0.001** |
| **HTN& elevated TG& low HDL-C, N (%)** | 11 (1.1) | 319 (5.9) | **<0.001** |
| **HTN& high WC& low HDL-C, N (%)** | 10 (1.0) | 259 (4.7) | **<0.001** |
| **Elevated TG& high WC& low HDL-C, N (%)** | 8 (0.8) | 187 (3.4) | **<0.001** |
| **Elevated glucose& HTN& elevated TG& high WC, N (%)** | 2 (0.2) | 107 (2.0) | **<0.001** |
| **Elevated glucose& HTN& elevated TG& low HDL-C, N (%)** | 3 (0.3) | 121 (2.2) | **<0.001** |
| **Elevated glucose& HTN& high WC& low HDL-C, N (%)** | 2 (0.2) | 95 (1.7) | **<0.001** |
| **Elevated glucose& elevated TG& high WC& low HDL-C, N (%)** | 1 (0.1) | 71 (1.3) | **<0.001** |
| **HTN& elevated TG& high WC& low HDL-C, N (%)** | 6 (0.6) | 146 (2.7) | **<0.001** |
| **All 5 metabolic components** | 1 (0.1) | 63 (1.2) | **<0.001** |
| **All 5 metabolic components& elevated hs-CRP** | 0 (0.0) | 30 (0.5) | **0.002** |
