# Supplementary material for: Transitioning from having no metabolic abnormality nor obesity to metabolic impairment in a cohort of apparently healthy adults
Source: Cardiovasc Diabetol. 2023 Aug 26;22:226. doi: 10.1186/s12933-023-01954-w (PMC10463945; doi:10.1186/s12933-023-01954-w)
Supplement: Supplementary file 5 — Additional file 5: Table S5. Deltas of biomarkers presented as mean and IQR in metabolic health and transition to metabolic impairment. Comparison of the group that remained healthy on both visits and the group that became metabolically impaired as measured on the 2nd visit. [file 12933_2023_1954_MOESM5_ESM.docx]

**Table S5: Deltas of biomarkers presented as mean and IQR in metabolic health and transition to metabolic impairment.**

Comparison of the group that remained healthy on both visits and the group that became metabolically impaired as measured on the 2^nd^ visit.

|  | **Healthy on both visits** | | **Healthy on visit 1; not healthy visit 2** | | **P-value (T-test)** |
| --- | --- | --- | --- | --- | --- |
|  | **Mean (SD)** | **Median, [IQR]** | **Mean (SD)** | **Median [IQR]** |  |
| **Delta Hs-CRP, mg/dL** | -0.1 (3.1) | 0.0 [-0.6-0.4] | 0.0 (4.8) | 0.0 [-0.4-0.8] | 0.802 |
| **Delta PLT (**${\boldsymbol{x}\boldsymbol{10}}^{\boldsymbol{3}}$**/µL)** | -6.3 (37.1) | -5.0 [-27.0-14.0] | -3.7 (33.7) | -4.0 [-24.0-16.0] | 0.145 |
| **Delta WBC,** ${\boldsymbol{x}\boldsymbol{10}}^{\boldsymbol{3}}$**/µL** | 0.0 (1.2) | 0.0 [-0.7-0.8] | 0.2 (1.3) | 0.1 [-0.5-0.8] | **0.033** |
| **Delta ALT, U/L** | 1.5 (11.8) | 1.0 [-3.0-6.0] | 3.1 (11.9) | 2.0 [-3.0-7.0] | **0.009** |
| **Delta AST, U/L** | 0.9 (9.0) | 1.0 [-3.0-4.0] | 1.7 (8.1) | 1.0 [-2.0-5.0] | 0.096 |
| **Delta GGT, U/L** | 4.9 (9.4) | 4.0 [1.0-7.0] | 5.8 (12.0) | 5.0 [1.0-9.0] | 0.077 |
| **Delta globulin** | -0.7 (2.5) | -1.0 [-2.0-1.0] | -0.6 (2.5) | -1.0 [-2.0-1.0] | 0.490 |
| **Delta LDH, U/L** | 12.3 (46.5) | 14.0 [-15.0-41.0] | 17.9 (53.6) | 16.0 [-11.0-45.0] | **0.031** |
| **Delta ALP, U/L** | 5.0 (14.0) | 5.0 [-3.0-12.0] | 7.0 (14.7) | 7.0 [-1.0-14.0] | **0.009** |
| **Delta creatinine,** | 0.0 (0.1) | 0.0 [-0.1-0.0] | 0.0 (0.1) | 0.0 [-0.1-0.1] | 0.761 |
| **Delta Uric Acid, mg/dL** | 0.0 (0.7) | 0.0 [-0.5-0.4] | 0.1 (0.8) | 0.0 [-0.4-0.5] | **0.018** |
| **Delta Sodium, mmol/L** | -0.3 (3.3) | 0.0 [-2.0-2.0] | -0.7 (3.3) | -1.0 [-3.0-1.0] | 0.066 |
| **Delta potassium, mmol/L** | 0.0 (0.5) | 0.0 [-0.3-0.3] | 0.0 (0.4) | 0.0 [-0.3-0.3] | 0.290 |
| **Delta LDL-C, mg/dL** | -0.9 (21.2) | 0.0 [-12.0-13.0] | -2.1 (24.3) | 0.0 [-11.0-14.0] | 0.278 |
| **Delta Bilirubin, mg/dL** | 0.0 (0.3) | 0.0 [-0.2-0.1] | -0.1 (0.3) | 0.0 [-0.2-0.1] | **0.016** |
| **Delta RBC,** $\boldsymbol{x}\boldsymbol{10}^{\boldsymbol{6}}$**/µL** | -0.1 (0.2) | -0.1 [-0.2-0.1] | 0.0 (0.2) | 0.0 [-0.2-0.1] | **0.004** |
| **Delta BMI** | -0.2 (2.1) | 0.0 [-0.9-0.9] | 0.1 (3.1) | 0.4 [-0.7-1.6] | **0.005** |
