# Supplementary material for: Transitioning from having no metabolic abnormality nor obesity to metabolic impairment in a cohort of apparently healthy adults
Source: Cardiovasc Diabetol. 2023 Aug 26;22:226. doi: 10.1186/s12933-023-01954-w (PMC10463945; doi:10.1186/s12933-023-01954-w)
Supplement: Supplementary file 6 — Additional file 6: Table S6. Univariate regression for metabolic impairment. The model includes delta values of biomarkers alongside metabolic and demographic components from the first visit. [file 12933_2023_1954_MOESM6_ESM.docx]

**Table S6**: **Univariate regression for metabolic impairment.**

The model includes delta values of biomarkers alongside metabolic and demographic components from the first visit.

| **Variable** | **P-value** | **OR** | **CI** |
| --- | --- | --- | --- |
| **Age (first visit), years** | **<0.001** | **1.029** | 1.020-1.038 |
| **Gender (males)** | **<0.001** | **1.457** | 1.229-1.729 |
| **Currently/ previously smoking** | 0.359 | 1.091 | 0.905-1.316 |
| **Deltas of Biomarkers** | | | |
| **Delta fibrinogen, g/L** | 0.397 | 0.999 | 0.996-1.002 |
| **Delta Creatinine mg/dL** | 0.761 | 0.861 | 0.327-2.264 |
| **Delta Albumin, g/L** | 0.811 | 1.005 | 0.966-1.045 |
| **Delta BUN, mg/dL** | 0.746 | 0.995 | 0.965-1.026 |
| **Delta PLT,** ${\boldsymbol{x}\boldsymbol{10}}^{\boldsymbol{3}}$**/µL** | 0.145 | 1.002 | 0.999-1.005 |
| **Delta RBC,** $\boldsymbol{x}\boldsymbol{10}^{\boldsymbol{6}}$**/µL** | **0.004** | **1.828** | 1.213-2.755 |
| **Delta WBC,** ${\boldsymbol{x}\boldsymbol{10}}^{\boldsymbol{3}}$**/µL** | **0.034** | **1.089** | 1.006-1.179 |
| **Delta Hemoglobin, g/dL** | 0.167 | 1.099 | 0.961-1.258 |
| **Delta Bilirubin, mg/dL** | **0.017** | **0.645** | 0.449-0.925 |
| **Delta AST U/L** | 0.099 | 1.010 | 0.998-1.022 |
| **Delta ALT U/L** | **0.010** | **1.012** | 1.003-1.020 |
| **Delta Uric acid, mg/dL** | **0.019** | **1.173** | 1.027-1.340 |
| **Delta Globulin, g/L** | 0.490 | 1.014 | 0.974-1.056 |
| **Delta ALP U/L** | **0.010** | **1.010** | 1.002-1.017 |
| **Delta LDH U/L** | **0.032** | **1.002** | 1.000-1.004 |
| **Delta GGT U/L** | 0.082 | 1.009 | 0.999-1.019 |
| **Delta Protein total, g/L** | 0.538 | 1.009 | 0.981-1.036 |
| **Delta Total Cholesterol mg/dL** | 0.742 | 0.999 | 0.996-1.003 |
| **Delta LDL mg/dL** | 0.284 | 0.998 | 0.993-1.002 |
| **Delta Chloride, mmol/L** | 0.270 | 0.980 | 0.945-1.016 |
| **Delta Potassium, mmol/L** | 0.290 | 0.885 | 0.706-1.110 |
| **Delta Calcium, mg/dL** | 0.836 | 0.980 | 0.813-1.182 |
| **Delta Sodium, mmol/L** | 0.066 | 0.972 | 0.942-1.002 |
| **Delta Phosphorus, mg/dL** | 0.996 | 0.999 | 0.802-1.245 |
| **Delta Hs-CRP, mg/dL** | 0.803 | 1.003 | 0.978-1.029 |
| **Delta FEV/FCV** | 0.364 | 1.008 | 0.990-1.027 |
| **Metabolic Variables on the first visit** | | | |
| **Diastolic, mmHg** | **<0.001** | **1.036** | 1.021-1.052 |
| **Systolic, mmHg** | **<0.001** | **1.023** | 1.013-1.033 |
| **BMI, kg/**$\boldsymbol{m}^{\boldsymbol{2}}$ | **<0.001** | **1.178** | 1.139-1.219 |
| **Waist Circumference, cm** | **<0.001** | **1.039** | 1.030-1.049 |
| **FPG, mg/dL** | **<0.001** | **1.038** | 1.025-1.052 |
| **HemA1C (%)** | **<0.001** | **2.123** | 1.470-3.066 |
| **Triglycerides, mg/dL** | **<0.001** | **1.007** | 1.004-1.010 |
| **HDL-C, mg/dL** | **<0.001** | **0.983** | 0.977-0.990 |
| **LDL-C, mg/dL** | **<0.001** | **1.007** | 1.004-1.010 |
| **Log(hs-CRP), mg/dL** | **<0.001** | **1.447** | 1.202-1.743 |
